# The C-type lectin receptor Dcir (*Clec4a2*) restrains *Aspergillus fumigatus* elimination by limiting the degranulatory activity of neutrophils

**DOI:** 10.3389/fimmu.2025.1639400

**Published:** 2025-08-04

**Authors:** Fabio Seiti Yamada Yoshikawa, Rikio Yabe, Shota Torigoe, Sho Yamasaki, Shinobu Saijo

**Affiliations:** ^1^ Division of Molecular Immunology, Medical Mycology Research Center, Chiba University, Chiba, Japan; ^2^ Cancer Immunology Project, Department of Diseases & Infection, Tokyo Metropolitan Institute of Medical Science, Tokyo, Japan; ^3^ Laboratory of Molecular Immunology, Immunology Frontier Research Center, Osaka University, Osaka, Japan; ^4^ Department of Mycobacteriology, Leprosy Research Center, National Institute of Infectious Diseases, Tokyo, Japan; ^5^ Research Center for Biosafety, Laboratory Animal and Pathogen Bank, National Institute of Infectious Diseases, Tokyo, Japan; ^6^ Department of Molecular Immunology, Research Institute for Microbial Diseases, Osaka University, Osaka, Japan; ^7^ Center for Infectious Disease Education and Research (CiDER), Osaka University, Osaka, Japan

**Keywords:** Dcir, Clec4a2, *Aspergillus fumigatus*, neutrophils, innate immunity

## Abstract

**Introduction:**

C-type lectin receptors (CLRs) are innate sensors crucial for antifungal and antimycobacterial responses, contributing to host defenses against pathogens, including the ubiquitous mold *Aspergillus fumigatus*. Dendritic cell immunoreceptor (Dcir) modulates immune responses by limiting the development of inflammation and autoimmunity; however, its involvement in fungal infections has not been previously established.

**Methods:**

Wild-type and Dcir-knockout C57BL/6J mice were infected with *A. fumigatus* intratracheally to establish a model of pulmonary aspergillosis. For *in vitro* analysis, neutrophils were purified from the bone marrow and incubated with *A. fumigatus* hyphae.

**Results:**

Mice lacking Dcir exhibited improved clearance of *A. fumigatus* from the lungs, while tissue inflammation—assessed by phagocyte recruitment and inflammatory cytokine levels within the lungs—did not change significantly compared to Dcir competent mice. Neutrophils from Dcir-deficient mice exhibited enhanced killing of *A. fumigatus* hyphae, attributed to higher degranulatory activity, triggered by intracellular Ca^2+^ mobilization.

**Discussion:**

The results indicate a potential association between Dcir and downregulation of signalling pathways associated with neutrophil exocytosis. Thus, Dcir is a potential novel fungal sensor that, unlike other CLR family members, primarily fine-tunes host effector responses.

## Introduction

*Aspergillus fumigatus* is an environmental mold with pathogenic potential, implicated in various clinical manifestations, ranging from mild allergic responses to severe, life-threatening diseases (1). Although individuals are routinely exposed to *Aspergillus* spores, most remain asymptomatic due to the immune system’s ability to facilitate fungal clearance while preventing immune overactivation (2). Nevertheless, the precise mechanisms underlying these protective responses have not been fully elucidated.

C-type lectin receptors (CLRs) are essential innate immune sensors involved in antifungal defense (3). CLRs activate various effector mechanisms necessary for protecting against *A. fumigatus*. For instance, dectin-1 (*CLEC7A* in humans and *Clec7a* in mice) regulates CD4^+^ T helper cell polarization (4), interleukin (IL)-17A/IL-22-driven neutrophil recruitment (5), and NK cell-dependent control of *A. fumigatus* via IL-15 release (6). Dectin-2 (*CLEC6A/Clec4n*) triggers extracellular trap production by plasmacytoid dendritic cells (7), whereas MelLec (*CLEC1A*/*Clec1a*) mediates fungal melanin recognition by endothelial cells (8).

Most CLRs use an immunoreceptor tyrosine-based activation motif (ITAM) for kinase activation and signal transduction. In contrast, Dendritic cell immunoreceptor (Dcir; *CLEC4A*/*Clec4a2*) signals through an immunoreceptor tyrosine-based inhibitory motif (ITIM), recruiting phosphatases and negatively regulating other signaling pathways (9). Hence, Dcir primarily has immunoregulatory functions, particularly in bone and joint health (10–12), while its deficiency has been linked to spontaneous autoimmune responses (13).

In the area of infectious diseases, Dcir is primarily associated with viral infections, such as HIV, where it acts as an attachment factor on dendritic cells and facilitates CD4+ T cell invasion (14). Furthermore, Dcir limits the induction of cytotoxic CD8^+^ T cells against the neurotropic Theiler’s murine encephalomyelitis virus, worsening brain injury (15), with similar effects in cerebral malaria caused by the parasite *Plasmodium falciparum* (16). In fungal infections, CLEC4A recognizes *Pneumocystis* spp (17); however, its effector function remains unclear. Thus, despite potential roles for Dcir in anti-fungal responses, further investigation is required to delineate the associated pathways.

We hypothesized that Dcir participates in defense against *A. fumigatus*. Our findings suggest that Dcir modulates the antifungal response, limiting *A. fumigatus* clearance. Mechanistically, Dcir dampens the degranulatory activity of neutrophils without altering cell recruitment or inducing tissue inflammation. The findings of this study reveal a novel immunomodulatory function for Dcir and reinforces its role as a regulator of host homeostasis.

## Materials and methods

### Mice

Dcir-deficient (*Clec4a2* ^–/–^) mice on a C57BL/6J background (13) and wild-type C57BL/6J (WT) mice (CLEA Japan, Tokyo, Japan) were co-housed for at least one week before beginning the study. Male and female mice were used, with no observed sex bias in the results. Mice were maintained under specific-pathogen-free conditions with a gamma-sterilized diet and acidified tap water (0.002 N HCl) *ad libitum*.

All experiments complied with the “Fundamental Guidelines for Proper Conduct of Animal Experiments and Related Activities in Academic Research Institutions under the Jurisdiction of the Ministry of Education, Culture, Sports, Science, and Technology” (Ministry of Education, Culture, Sports, Science and Technology, Japan, 2006). The Institutional Animal Care and Use Committee from Chiba University approved all protocols (process number: A5-204).

### Fungal strains and inoculum preparation

*Aspergillus fumigatus* MYA4609 (strain CBS 101355 [AF 293], American Type Culture Collection, VA, USA) was maintained in Sabouraud Dextrose Agar (BD, NJ, USA) at 30°C with weekly subcultures. Conidia and germinating hyphae were generated as described by Yoshikawa et al. (6).

### Pulmonary aspergillosis model

To induce pulmonary aspergillosis, sedated mice had their trachea accessed orally using a 20Gx 1 1/4” indwelling needle intravenous cannula (Terumo, Tokyo, Japan). Mice then passively inhaled 1 x 10^7^ conidia cells in 30 μL of phosphate buffer saline (PBS).

To deplete neutrophils, mice were intravenously treated with 200 μg of anti-Ly6G antibody (Selleck Biotechnology, Kanagawa, Japan) or an isotype control (Rat IgG2a, κ, Selleck Biotechnology, Kanagawa, Japan) one day before infection.

To inhibit neutrophil degranulation *in vivo*, mice received intraperitoneal Nexinhib 20 (Tocris Bioscience, Bristol, United Kingdom) on days 1 and 3 post-infection (30 mg/kg), as previously described (18). At specified post-infection days, the mice were sedated and euthanized; bronchoalveolar lavage fluid (BALF) was collected via tracheal injection of 1 mL ice-cold PBS. Recovered cells underwent flow cytometric analysis (FCM, see section below). Lungs were harvested and processed according to the experiment’s objective.

#### Fungal burden assessment

Lungs were weighed, macerated in PBS, and the suspensions were used to measure colony-forming units (CFU) and cytokines. For CFU determination, dilutions were seeded in potato dextrose agar (PDA) plates, and colonies recovered after a 3-day incubation at 30°C were counted.

#### Histopathological analyses

Lungs were perfused with PBS and fixed overnight in formalin solution (Fujifilm Wako, Osaka, Japan). Samples were paraffinized, and sections were stained with Grocott’s methenamine silver.

#### Immunophenotyping of the cellular infiltrate

Lungs were digested with Collagenase IV buffer (150 U/mL in RPMI-1640 medium; Sigma-Aldrich, Germany) using a protocol adapted from D’Agostino et al. (19). Recovered cells were stained for FCM.

### Fc chimeras and binding analysis

The Dcir-fused Fc protein chimera was generated as previously described (6). The extracellular domain of Dcir was bound to the Fc portion of human IgG2 in a pIRES bleo3 vector plasmid; a vector lacking the CLR insert served as the control. Binding of Dcir to *A. fumigatus* isoforms was evaluated using an enzyme-linked immunosorbent assay (ELISA)-like assay, as previously described (6). Optical density (OD) measurements were used to construct binding curves for nonlinear regression analysis.

### Bone marrow neutrophil assays

Neutrophils were isolated from the bone marrows of WT and *Clec4a2* ^—/—^ mice using Percoll gradient centrifugation as previously described (6). Neutrophil purity (> 90%) and Dcir expression were analyzed by FCM.

Neutrophils were plated into 96-well plates containing RPMI-1640 medium (Fujifilm Wako, Osaka, Japan) supplemented with 10% inactivated fetal bovine serum (iFBS; Biosera, Nuaillé, France). Cells were incubated with *A. fumigatus* isoforms at 37°C and 5% CO_2_ for up to 3 h at a ratio of five neutrophils per fungal cell. Cell-free supernatants were collected and stored at –80°C for subsequent cytokine and Lipocalin-2 quantification using a commercial Mouse Lipocalin-2/NGAL DuoSet ELISA kit (R&D systems, MN, USA), or used immediately in other assays.

The remaining neutrophils were lysed with 1% Triton X-100 solution, prepared in-house from polyethylene glycol mono-p-isooctylphenyl ether (Nacalai Tesque, Kyoto, Japan). The number of surviving conidia was determined by seeding on PDA plates for CFU enumeration. Hyphal viability was assessed via the 3-[4,5-dimethylthiazol-2-yl]-2,5 diphenyl tetrazolium bromide (MTT) assay.

For degranulation inhibition, upon addition of *A. fumigatus* to neutrophil cultures, wells were supplemented with 1 μM of Nexinhib 20 (Tocris Bioscience, Bristol, United Kingdom) or DMSO as a vehicle control.

For microscopy, cells were seeded onto glass slides in 24-well plates and incubated with *A. fumigatus* under the same conditions described above. Slides were stained using the Neat Stain Hematological Stain kit (Polysciences Inc., PA, USA), and images were acquired via optical microscopy.

### Cell viability

For assessment of cell viability, the Sytox Green incorporation assay was employed (20). PMA (phorbol 12-myristate 13-acetate; 5 μg/mL) was used as a positive control. Fluorescence in the FITC channel was measured with FCM, and the percentage of positive cells was recorded.

### Reactive oxygen species measurements

Intracellular ROS levels were measured using a commercial DCFDA/H2DCFDA-Cellular ROS Assay Kit (Abcam, Cambridge, UK) according to the manufacturer’s instructions. Fluorescence was detected via FCM (FITC channel).

For extracellular ROS measurement, a cytochrome C reduction assay was performed to measure superoxide (O_2_^-^) in the supernatants as previously described (21). Neutrophils were plated in 96-well plates with 10% iFBS in HBSS and incubated with *A. fumigatus* or 100 nM PMA. The cell-free supernatants were combined with 1 mg/mL cytochrome C, with or without 100 U/mL superoxide dismutase (SOD) (stock solutions prepared in HBSS; Nacalai Tesque, Kyoto, Japan). Absorbance was recorded at 550, 540, and 560 nm. The OD at 550 nm was adjusted by the average OD between 540 and 560 nm (ODcorr) for calculations. The O_2_^-^concentration was calculated using the formula:


$$O_{2}^{-}=\;\left(\left[OD\;without\;SOD\right]–\left[OD\;with\;SOD\right]\right)/21.1\;mmol/L^{-1}cm^{-1}$$

where the constant 21.1 is the extinction coefficient for reduced cytochrome C; results are expressed as mmol/μL.

### Zymography

Gelatinase activity in neutrophil cultures was assessed via zymography. Cell-free supernatants from *A. fumigatus*-stimulated neutrophil cultures were separated by sodium dodecyl sulfate–polyacrylamide gel electrophoresis (SDS–PAGE) using an in-house 6% polyacrylamide gel with 1 mg/mL gelatin under non-reducing and non-denaturing conditions. Gels were renatured (Zymogram Renaturing Buffer, Novex, CA, USA), developed overnight at 37°C (Zymogram Developing Buffer, Novex), and stained with Bio-Safe Coomassie G-250 stain (Bio-Rad, USA). Digested regions appeared as clear bands on a blue background.

### Western blot

For MMP9 detection, supernatants were run on SDS-PAGE (non-reducing, non-denaturing), transferred to polyvinylidene difluoride (PVDF) membranes (Bio-Rad, USA), and analyzed by western blot. For PLCγ2/pPLCγ2 detection, neutrophil lysates underwent SDS-PAGE (reducing, denaturing) before transfer to PVDF membranes. Primary antibodies included: Rb mAb to MMP9 (Abcam, MA, USA), PLCgamma2 Rabbit Ab, phospho-PLCγ2 (Tyr1217) Ab (Cell Signaling, MA, USA), and anti-β-actin pAb (MBL, Tokyo, Japan). HRP donkey anti-rabbit IgG (Biolegend, CA, USA) served as the secondary antibody. Zymography and western blot images were quantified using ImageJ (version 1.53 for Mac OS X, NIH, USA).

### Intracellular Ca^2+^ measurements

Intracellular Ca^2+^ levels were estimated by FCM using the fluorescent probe 4-Fluo AM (Dojindo, Tokyo, Japan). After stimulation with *A. fumigatus*, mean fluorescence intensity (MFI) values were recorded using FCM (Fluo-4AM) in the FITC channel.

### Immunophenotyping/FCM

For cell phenotyping, cells were stained for FCM analysis as previously described. Reagents and antibodies are listed in **Supplementary Table 1**. Data were acquired with a FACS Verse flow cytometer (8-color, BD, NJ, USA) and analyzed using FlowJo (v.10.7.1 for Mac OS X, BD, NJ, USA). Gating strategies are shown in **Supplementary Figure 3**.

### Cytokine measurements

Cytokines were measured with the BD Cytometric Bead Array assay per the manufacturer’s instructions. Data were acquired on a FACS Verse and analyzed using FCAP Array software (v.3.0.1; BD). Detection limits were as follows: IL-1β,  1.9 pg/mL; IL-6, 1.4 pg/mL; TNF, 2.8 pg/mL; IL-10, 9.6 pg/mL; MCP-1, 2.7 pg/mL; KC, 0.1 pg/mL; CCL3/MIP-1α, 2.3 pg/mL; and CCL4/MIP-1β, 0.6 pg/mL.

### Statistical analysis

Statistical analyses were conducted using GraphPad Prism (v. 10 for OSX, GraphPad Inc., CA, USA). Data were screened for outliers using the ROUT method, and group comparisons were made using non-parametric tests, regardless of normality results. The statistical test employed for each analysis, sample size, and number of replicates are disclosed in the figure legends. A *p-*value < 0.05 was considered statistically significant.

## Results

### Dcir limits *A. fumigatus* clearance

In a previously described model of acute disseminated aspergillosis, immunocompetent WT mice were shown to develop transient, self-limiting disease, while dectin-1 knockout mice readily succumbed to the infection (6), facilitating the rapid screening of susceptibility factors.

We employed this model to initially assess the potential contribution of Dcir to antifungal defense (**Supplementary Figure 1**). *Clec4a2* ^—/—^ mice experienced no mortality, less weight loss (**Supplementary Figure 1A**) (area under the curve [AUC]: mean 47.22 vs. 17.51, *p =* 0.0279), and lower spleen *A. fumigatus* burden than WT mice (**Supplementary Figure 1B**; 5 days post infection [dpi] mean 106360 vs. 45259, *p =* 0.0377), with only IL-1β levels markedly reduced among cytokines (**Supplementary Figure 1C**). These results suggest that Dcir restricts pathogen clearance rather than promoting anti-fungal responses.

**Figure 1:**
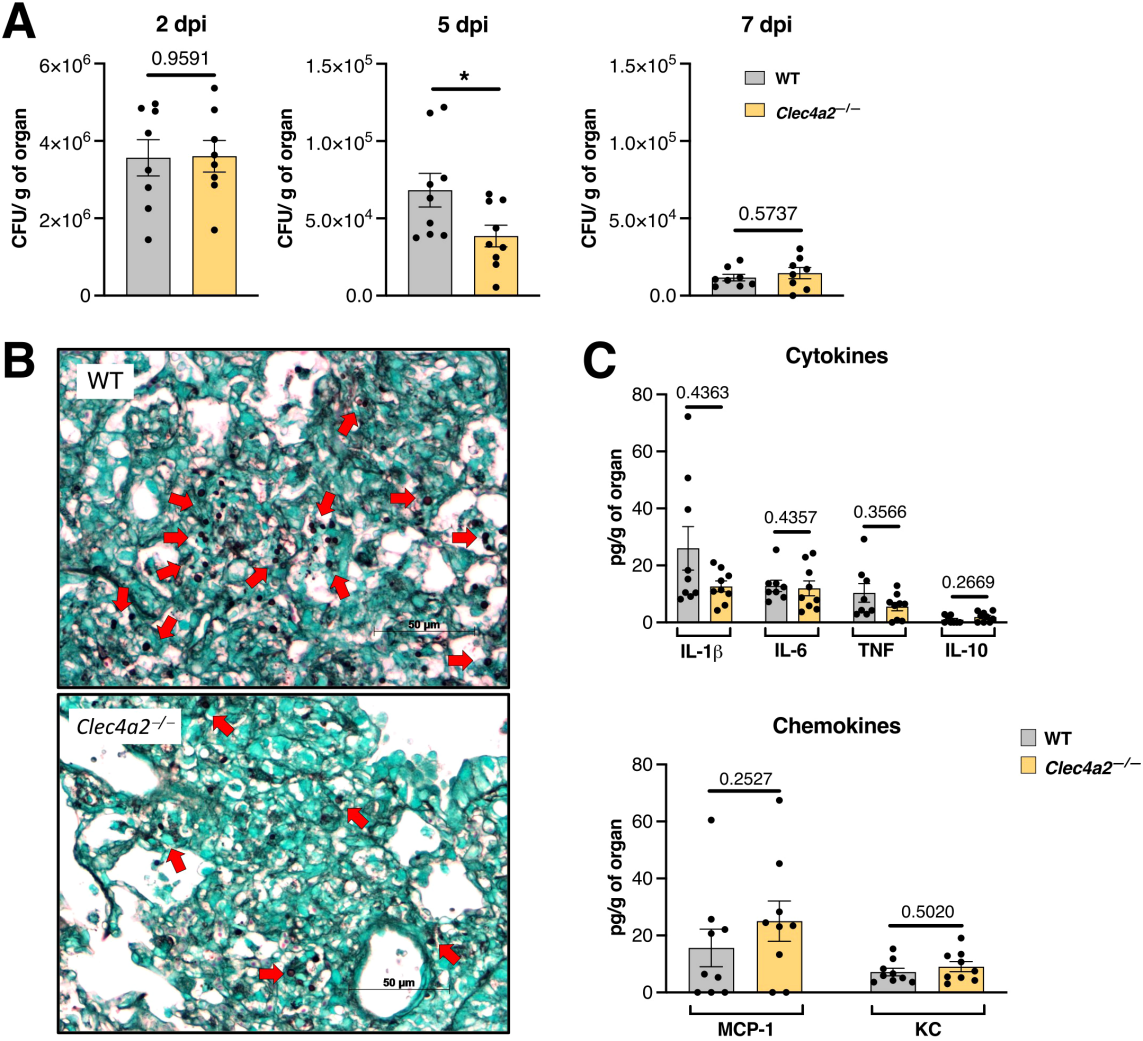
Dcir-deficient mice display improved anti-*Aspergillus* clearance. **(A)** WT and *Clec4a2* ^—/—^ mice were infected intratracheally with 1 x10^7^ *A. fumigatus* conidia, and the fungal burden in lung macerates was determined; *N* = 8–9 mice per group, pooled from two independent experiments. **(B)** Silver-stained lung sections from WT and *Clec4a2* ^—/—^ mice harvested 5 days post-infection (dpi). Red arrows indicate fungal structures. Images are representative of three mice per group. **(C)** Cytokine and chemokine levels in lung macerates harvested 5 dpi; *N* = 9 mice per group, pooled from two independent experiments. **(A, C)** Data are presented as mean ± SEM (each dot represents one mouse): Mann–Whitney U-test: *p < 0.05.

Next, in a standard pulmonary aspergillosis model that more closely mimics natural infection, WT and *Clec4a2* ^—/—^ mice were intratracheally infected with *A. fumigatus* (**Figure 1**). Although WT and *Clec4a2* ^—/—^ mice both cleared the infection efficiently, the knockout group showed a lower fungal burden at mid-infection (5 dpi; **Figure 1A**; mean 68267 vs. 38604, *p =* 0.0315). This was also observed in lung sections harvested at the same time point and stained with Grocott (silver-based) stain (black structures against the green-stained lung tissue) in *Clec4a2* ^—/—^ mice (**Figure 1B**). However, pulmonary cytokine and chemokine levels were similar between groups (**Figure 1C**), suggesting equivalent inflammatory responses in WT and *Clec4a2* ^—/—^ mice. Overall, these results suggest that Dcir deficiency enhances fungal elimination.

### Dcir absence enhances the anti-*A. fumigatus* activity of neutrophils

Neutrophils are crucial for *Aspergillus* clearance (22) and neutrophil recruitment is altered in Dcir-deficient mice in experimental models of chemical hepatitis (23) and DSS-induced colitis (24). Thus, we investigated phagocyte recruitment by analyzing the phenotypes of innate immune cells within the lungs and BALF harvested 5 dpi.

**Figure 2:**
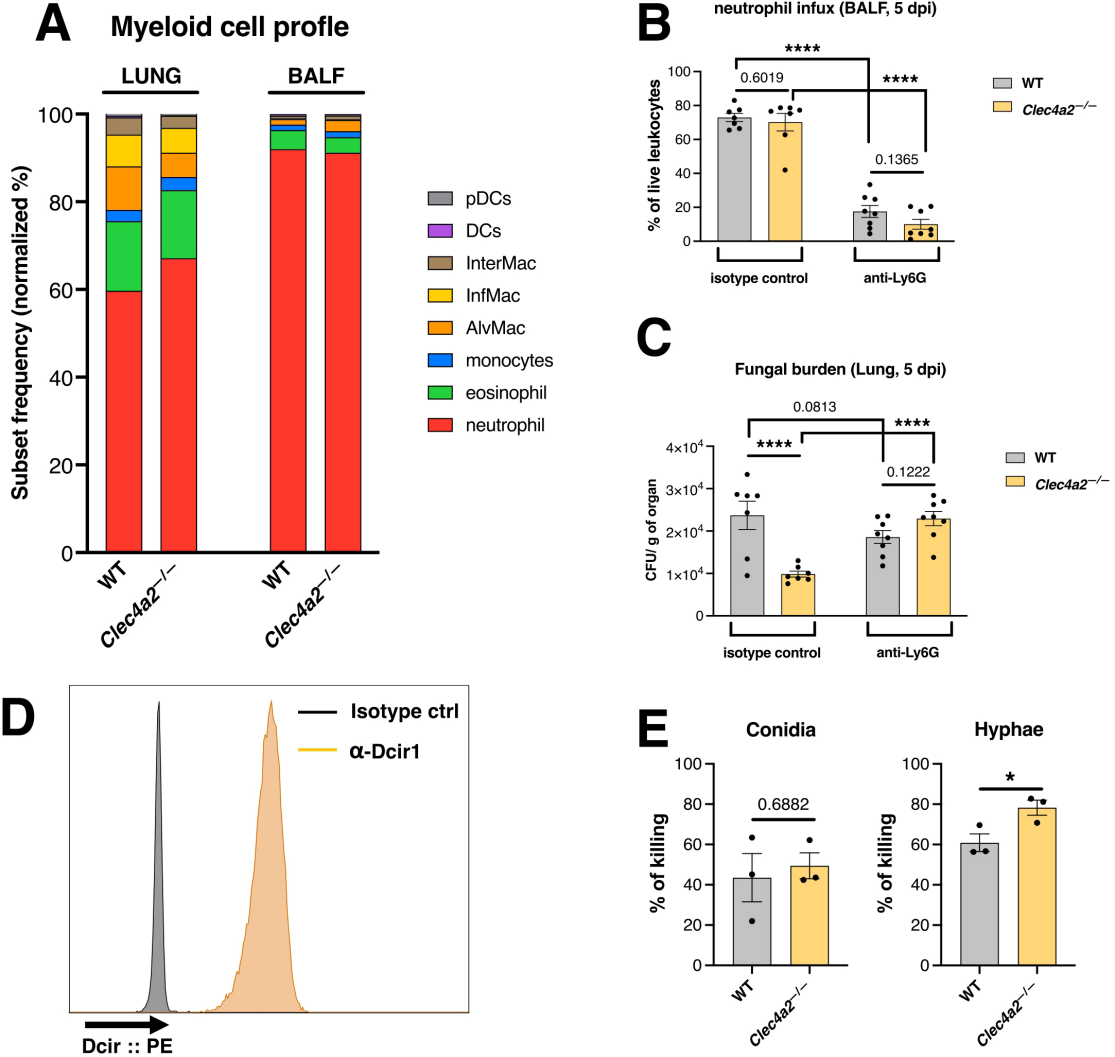
Dcir-deficient neutrophils display enhanced *A. fumigatus-*targeted killing. **(A)** Profile of innate cells recruited to the lungs and BALF of WT and *Clec4a2* ^—/—^ mice infected with 1 x10^7^ *A. fumigatus* conidia intratracheally and harvested 5 dpi. Data are presented as the normalized proportion calculated based on the cellular counts. **(B)** Recruitment of neutrophils in the BALF and **(C)** fungal burden in the lungs of animals treated with anti-Ly6G depleting antibody or isotype control and infected with *A. fumigatus* (samples harvested 5 dpi). **(B, C)** *N* = 7–8 mice per group, pooled from two independent experiments. Data are presented as mean ± SEM (each dot represents one mouse): Two-way ANOVA and Fisher’s LSD test: ****p < 0.0001. **(D)** Expression of Dcir on freshly isolated bone marrow neutrophils. Histogram data are representative of three independent experiments. **(E)** Neutrophil-mediated killing of *A. fumigatus* isoforms *in vitro*. Bone marrow-isolated neutrophils were incubated with *A. fumigatus* isoforms, and the surviving fungi were quantified using CFU (conidia) or MTT (hyphae) assays. Data are presented as mean ± SEM, pooled from three independent experiments: Unpaired *t*-test: *p < 0.05.

Neutrophils constituted the predominant cell population in the lung and BALF infiltrates of infected mice (**Figure 2A**), with no significant differences between WT and *Clec4a2* ^—/—^ mice (**Supplementary Figure 2**). While *Clec4a2* ^—/—^ mice exhibited a statistically significant reduction in alveolar macrophages (Mϕ), inflammatory Mϕ, and dendritic cells counts (**Supplementary Figure 2**), the frequencies of these subsets remained low, making up less than 10% of the total infiltrate. Thus, the response of *Clec4a2* ^—/—^ mice was not related to improved phagocyte recruitment.

Considering that neutropenia is a critical risk factor for aspergillosis (25, 26) and that neutrophils dominate the cellular infiltrates, we determined whether neutrophil-depleted *Clec4a2* ^—/—^ mice retained improved infection clearance compared to the WT group. Neutrophils were depleted using an anti-Ly6G antibody before infection (**Figure 2B**) and fungal burden was subsequently analyzed. Neutrophil depletion abolished the protective effect conferred by Dcir deficiency (**Figure 2C**; isotype: mean 23695 vs. 9850, *p*<0.0001; anti-Ly6G: mean 18574 vs. 22933, *p* = 0.1222). Additionally, Dcir was significantly expressed by neutrophils (**Figure 2D**), confirming previous findings (27, 28). These results suggest that Dcir directly regulates neutrophil effector functions against *A. fumigatus*.

**Figure 3:**
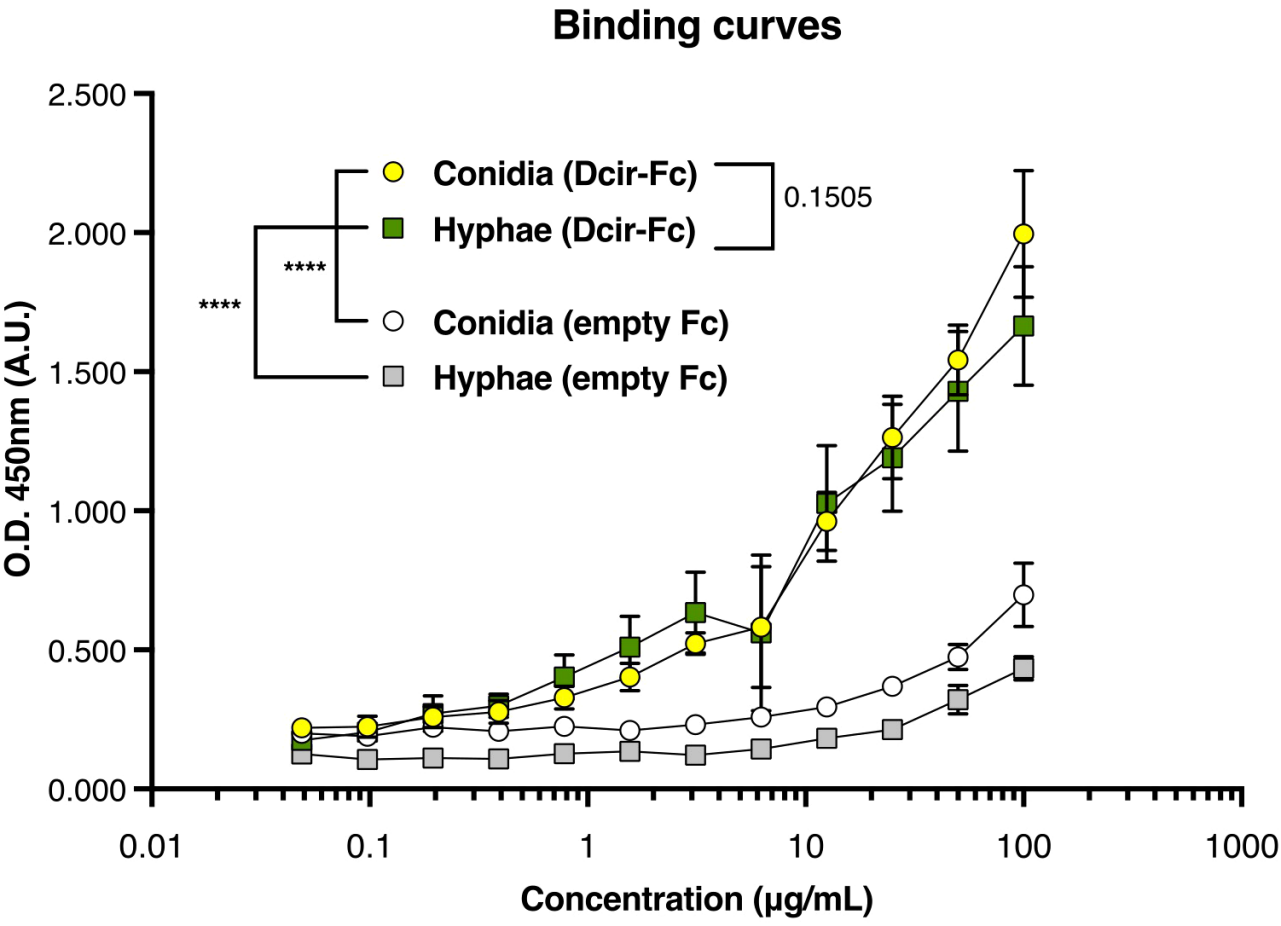
Dcir recognizes *A. fumigatus* conidia and hyphae. Fungal cells were stained with Dcir Fc chimeras, and binding was assessed using EIA assay. Data are presented as absorbance reads at 450 nm expressed as mean ± SEM, pooled from three independent experiments: Nonlinear regression: ****p < 0.0001.

To further investigate this mechanism, *in vitro* assays were conducted wherein bone marrow-derived neutrophils were incubated with different *A. fumigatus* isoforms to assess their phagocytic killing function. *Clec4a2* ^—/—^ neutrophils exhibited enhanced hyphal killing (**Figure 2E**; mean 60.88 vs. 78.27, *p =* 0.0391). However, the absence of Dcir did not uniformly enhance conidial killing. As *A. fumigatus* spores evade dectin-1 recognition due to hydrophobin A masking binding sites on the conidial surface (29), we explored whether a similar mechanism might affect Dcir sensing. Using a chimeric Dcir soluble protein comprising the receptor’s extracellular domain fused to a human IgG Fc fragment, we evaluated binding to *A. fumigatus* isoforms. Unexpectedly, Dcir bound conidia and hyphae (**Figure 3**), suggesting that evasion analogous to dectin-1 is not operative. These data support the hypothesis that, while Dcir binds both forms, it specifically modulates neutrophil effector functions against hyphae rather than conidia.

Therefore, neutrophils from *Clec4a2* ^—/—^ mice eliminated fungi more effectively, likely explaining their enhanced clearance capability, despite unchanged tissue inflammation or cell recruitment.

### Supernatants from *Clec4a2* ^—/—^ neutrophils display higher enzymatic activity

Next, the effector mechanisms through which Dcir regulates fungicidal activity against *A. fumigatus* were investigated. Neutrophils eliminate pathogens via phagocytosis, programmed cell death (NETosis), oxidative stress, and degranulation (30). Given the size disparity between neutrophils and *A. fumigatus* hyphae, phagocytosis was ruled out and we focused on the other possibilities.

**Figure 4:**
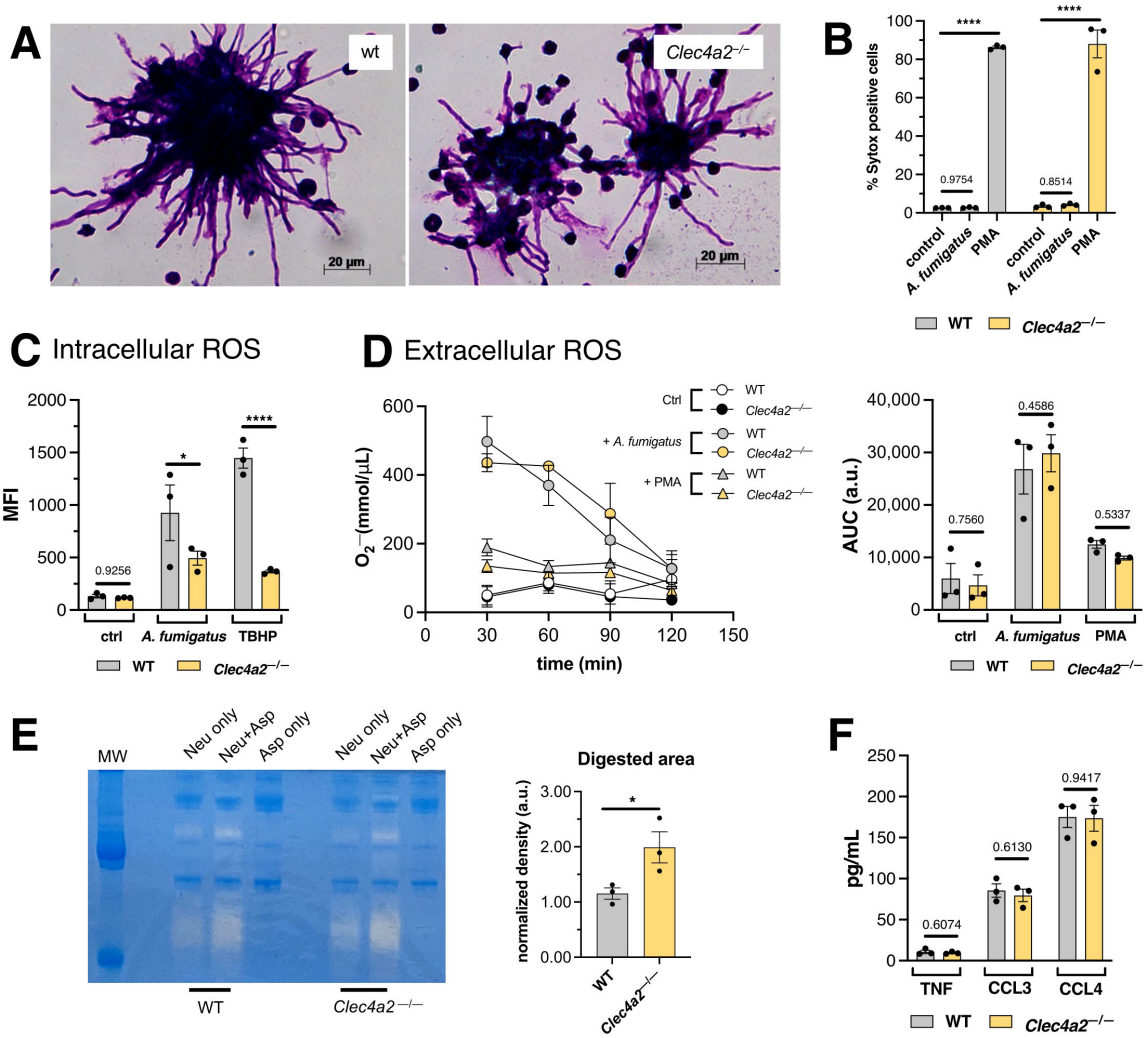
Dcir alters the release of gelatinases by neutrophils. **(A)** Photomicrographs depicting the interaction between WT and *Clec4a2* ^—/—^ neutrophils with *A. fumigatus*. Images are representative of three independent experiments. **(B)** Assessment of DNA release using SYTOX green incorporation assay. **(C)** Intracellular ROS production by neutrophils incubated with *A. fumigatus* determined using the DCFDA conversion assay. **(D)** O_2_^-^ production curves of extracellular ROS production by neutrophils incubated with *A. fumigatus*, determine using the cytochrome C assay; area under the curve (AUC) was calculated. **(E)** Zymography assay for gelatinase activity in supernatants from the *in vitro* cultures. **(F)** Cytokines measured in the supernatants of bone marrow neutrophils incubated with *A. fumigatus*. **(B–D)** Data are presented as mean ± SEM, pooled from three independent experiments. Two-way ANOVA and Fischer’s LSD posttest: *p < 0.05, ****p < 0.0001. **(E, F)** Data are presented as mean ± SEM, pooled from three independent experiments. Unpaired *t*-test: *p < 0.05.

Neutrophils accumulated around the hyphal structures (**Figure 4A**); however, significant cell death was not detected following *A. fumigatus* stimulation within our experimental time frame, as assessed by SYTOX probe incorporation (**Figure 4B**). Consequently, the increased killing of *A. fumigatus-*hyphae promoted by *Clec4a2* ^—/—^ cells (**Figure 2B**) appears to occur independently of neutrophil death.

The oxidative stress induced in *A. fumigatus*-stimulated neutrophils was assessed in sequence. Tokieda et al. (24) reported that *Clec4a2* ^—/—^ neutrophils exhibit impaired intracellular ROS production when exposed to lipopolysaccharide. In alignment with these findings, lower ROS production was observed in response to *A. fumigatus* and the positive control TBHP, in Dcir-deficient cells (**Figure 4C**). Although the underlying molecular mechanisms responsible for this intrinsic defect remain to be elucidated, its impact might be minimal in terms of fungal killing, given that even the inactivation of internalized conidia can also be achieved via non-oxidative mechanisms (31).

Subsequently, extracellular ROS production was also evaluated, which originates from a distinct source compared to intracellular ROS (32). Measurement of O_2_^-^ in culture supernatants using the cytochrome C assay revealed no significant difference between WT and *Clec4a2* ^—/—^ cells, despite the pronounced oxidative burst induced by *A. fumigatus* (**Figure 4D**). These findings suggest that oxidative stress does not account for the increased fungicidal activity observed in the knockout cells.

Interestingly, the supernatants from *A. fumigatus*-activated neutrophil cultures exhibited gelatinase activity, which was higher in Dcir-deficient cells (**Figure 4E**; mean 1.153 vs. 1.990, *p =* 0.0492). However, other secreted proteins, as cytokines, were not equally enhanced in the knockout cells (**Figure 4F**), suggesting that enzyme release is being independently favored. Neutrophil gelatinases are stored in granules and can be exocytosed sequentially (33), hinting that *Clec4a2* ^—/—^ neutrophils may exert increased degranulatory responses.

### *Clec4a2* ^—/—^ neutrophils display enhanced degranulation in response to *A. fumigatus*

**Figure 5:**
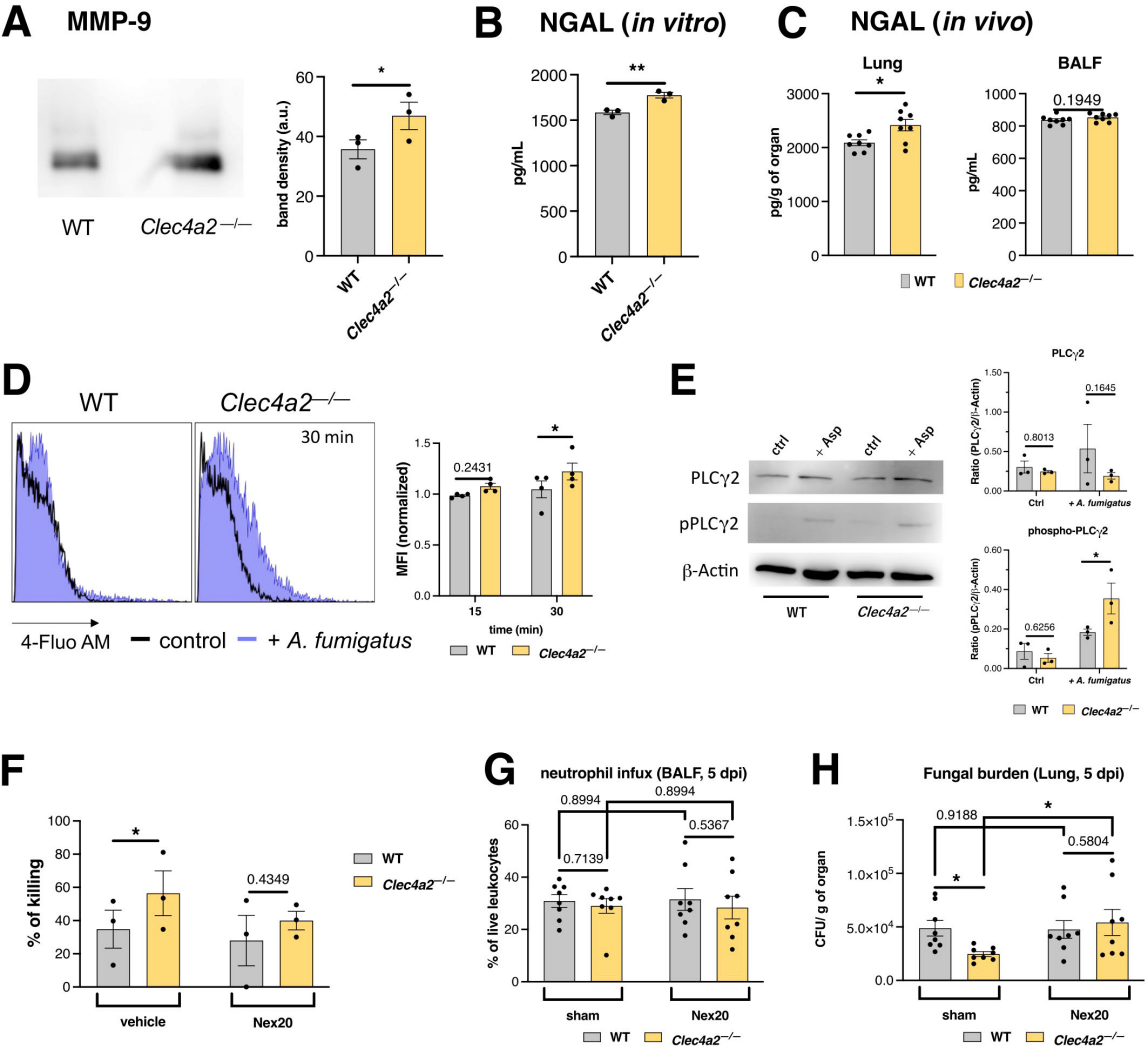
Dcir dampens *A. fumigatus*-induced neutrophil degranulation. **(A)** MMP-9 detection in the supernatants of *A. fumigatus*-stimulated neutrophil cultures using western blotting. Band density was calculated using ImageJ; data are presented as the mean ± SEM, pooled from three independent experiments. **(B)** Lipocalin-2/NGAL levels in the supernatants of *A. fumigatus*-stimulated neutrophil cultures; data are presented as mean ± SEM, pooled from three independent experiments. **(A, B)** Paired *t*-test: *p < 0.05, **p < 0.01. **(C)** Lipocalin-2/NGAL levels in the lungs and BALF of WT and *Clec4a2* ^—/—^ mice infected intratracheally with *A. fumigatus* conidia (5 dpi). Data are presented as mean ± SEM (each dot represents one mouse) pooled from two independent experiments. Mann–Whitney U-test: *p < 0.05. **(D)** Intracellular calcium mobilization estimated using the fluorescent probe Fluo-4AM; Left panel: representative histograms of Fluo-4AM fluorescence. Right panel: normalized MFI values presented as mean ± SEM pooled from three independent experiments. **(E)** PLCγ2 phosphorylation was analyzed using western blotting from *A. fumigatus*-stimulated neutrophil extracts. Left panel: representative blots of the analyzed proteins. Right panel: Band density was calculated in ImageJ; β-actin normalized values are presented as mean ± SEM, pooled from three independent experiments. **(F–H)** Pharmacological inhibition of degranulation with the compound Nexinhib 20. **(F)** *A. fumigatus*-stimulated neutrophils incubated with inhibitor, and *A. fumigatus* survival was assessed using the MTT assay. Data are presented as mean ± SEM, pooled from three independent experiments. **(G)** Neutrophil recruitment in BALF and **(H)** fungal burden in the lungs of animals treated with Nexinhib 20 (30 mg/kg) or sham, and infected with *A. fumigatus* (samples harvested 5 dpi). *N* = 8 mice per group. Data are presented as mean ± SEM pooled from two independent experiments (each dot represents one mouse). **(D–G)** Two-way ANOVA and Fischer’s LSD posttest: *p < 0.05.

To determine whether *Clec4a2* ^—/—^ neutrophils exhibit enhanced degranulation following *A. fumigatus* challenge, the levels of two proxy markers—metalloproteinase [MMP]-9 and Lipocalin-2/NGAL (34)—were quantified, confirming an increase in both markers in the supernatants of knockout cells (**Figure 5A**, mean 35.71 vs. 46.91, *p =* 0.0465; **Figure 5B**, mean 1585 vs. 1775, *p =* 0.0043). Notably, elevated NGAL concentrations were also detected in the lungs of infected *Clec4a2* ^—/—^ mice, supporting the *in vivo* relevance of the findings (**Figure 5C**; lung: mean 2090 vs. 2416, *p =* 0.0207).

Degranulation initiation is linked to increased cytoplasmic Ca^2+^, mobilized from intracellular stores (33). Using the fluorescent probe 4-Fluo AM, Ca^2+^ levels in *Clec4a2* ^—/—^ cells were monitored, revealing a more pronounced increase upon *A. fumigatus* stimulation compared to controls (**Figure 5D**; mean 1.048 vs. 1.223, *p =* 0.0499).

Various receptors can drive Ca^2+^ mobilization via their specific stimulatory pathways; however, a common signaling hub upstream of Ca^2+^ mobilization is phospholipase PLCγ2 phosphorylation/activation (35). *Clec4a2* ^—/—^ neutrophils displayed enhanced PLCγ2 phosphorylation after *A. fumigatus* stimulation (**Figure 5E**; pPLCγ2: mean 0.1837 vs. 0.3543, *p =* 0.0305). These data suggest that Dcir negatively regulates the signaling cascade leading to neutrophil degranulation.

To assess the importance of degranulation in *A. fumigatus* killing, this pathway was pharmacologically inhibited using Nexinhib 20—a neutrophil-specific drug (18). Inhibition of this pathway abolished the enhanced antifungal effect observed in Dcir-deficient neutrophils (**Figure 5F**; vehicle: mean 34.83 vs. 56.43, *p* = 0.0408; Nex20: mean 28.00 vs. 40.00, *p* = 0.4349). These findings were validated *in vivo*, wherein Nexinhib 20 was administered during infection. Despite the lack of changes in pulmonary neutrophil influx (**Figure 5G**), Nexinhib 20 treatment neutralized the advantage of Dcir knockout (**Figure 5H**; sham: mean 48782 vs. 24681, *p* = 0.0491; Nex20: mean 47576 vs. 54130, *p* = 0.5804), consistent with the effects observed following antibody-mediated neutrophil depletion (**Figure 2**).

Collectively, these results demonstrate that Dcir modulates the neutrophil-driven elimination of *A. fumigatus* by dampening the degranulation process.

## Discussion

**Figure 6:**
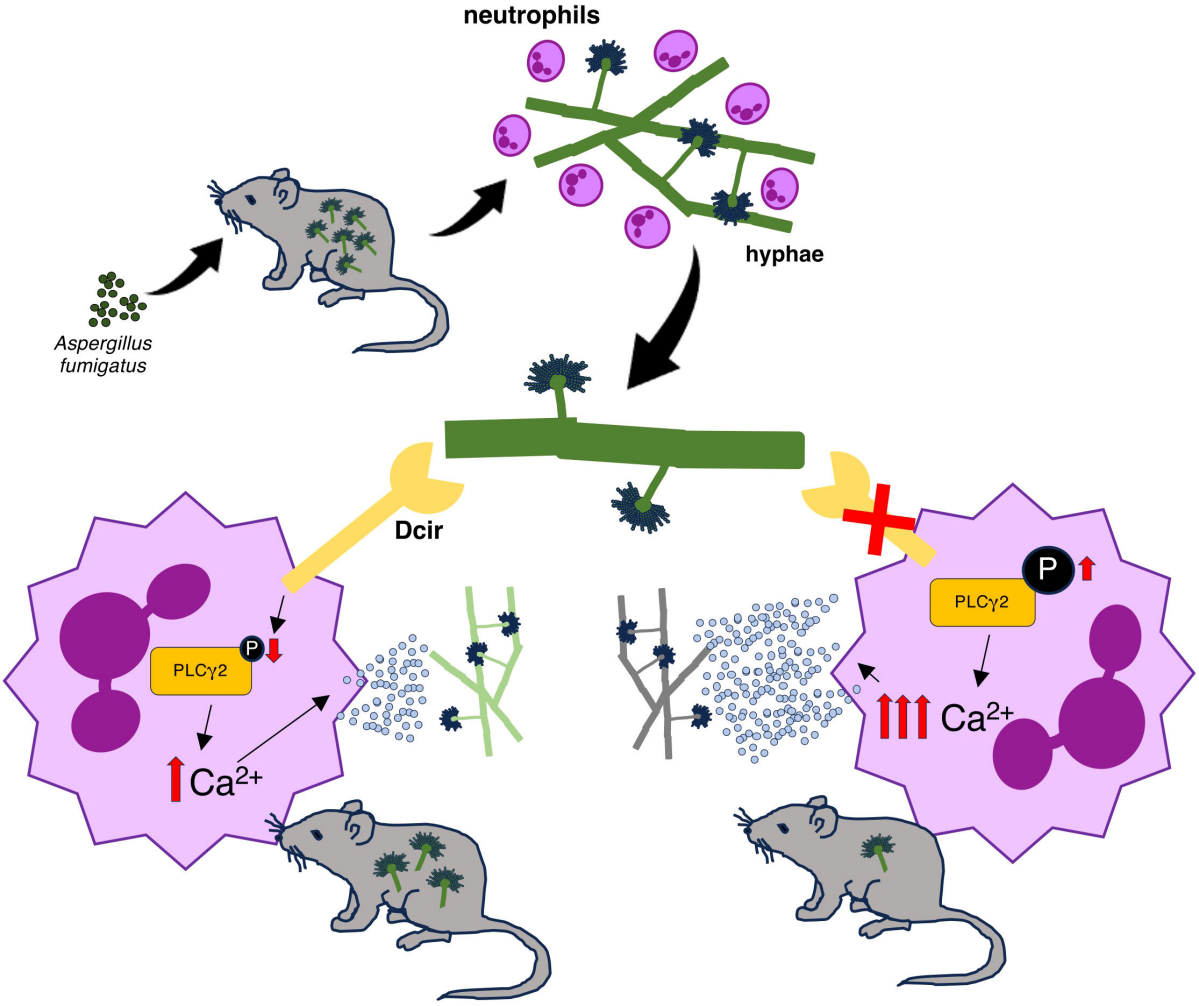
Proposed role for the regulatory function of Dcir in the host response to *A. fumigatus*. *A. fumigatus* infection promotes neutrophil recruitment, and Dcir recognizes the hyphae structures. Dcir-dependent signaling reduces PLCγ2 phosphorylation, which modulates the release of intracellular calcium, thereby limiting the release of neutrophil granules and delaying fungal clearance.

In fungal infections, CLRs usually act as inflammatory initiators that drive effector functions, promoting pathogen elimination. In the current study, Dcir was identified as a regulator of the anti-*Aspergillus* defense, limiting neutrophil degranulation and delaying the fungal clearance (**Figure 6**).

Richard et al. (28) postulated that Dcir regulates neutrophil activity but did not investigate beyond its expression in human cells. In the current study, neutrophil degranulation was identified as an essential effector mechanism for eliminating *A. fumigatus* hyphae. Dcir modulated this process by influencing granule release signaling—specifically, PLCγ2 phosphorylation and intracellular Ca^2+^ mobilization. This mechanism aligns with ITIM-based CLR modulation, where Dcir regulates pathway activation via dephosphorylation (9), and PLCγ2 was identified as a potential target of the Dcir-related phosphatase SHP2 (36).

Luo et al. showed that mast cells from Dcir-deficient mice exhibit lower degranulation in a cockroach allergen-induced atopic dermatitis model (37), contrasting with the current study’s results. However, the authors pointed that mast cell exocytosis depends on ROS-mediated oxidation of the intermediate protein calmodulin kinase II, and Dcir-deficient mast cells also have impaired intracellular ROS production—aligning with the current results. Therefore, Dcir’s role likely varies by model and cell type. Thus, data interpretation should account for these limitations.

Although *A. fumigatus* promotes NETosis (38), substantial cell death was not observed within the current experimental time window (**Figure 4B**). Thus, the involvement of NETosis was not investigated further. This observation may have been due to assay timing, as NET formation is a “late phase” neutrophil response that becomes detectable after more than six hours (39). Future studies should investigate the relationship between Dcir and NETs using under different experimental conditions.

Experimental aspergillosis self-resolves quickly in resistant mice, similar to human cases (1, 25). Therefore, the current study did not examine Dcir’s role in adaptive responses, particularly the antifungal T_H_17 profile. Previously we found that classical lymphocytes and IL-17 are not required for *A. fumigatus* defense in disseminated disease (6). However, several other aspergillosis models depend on the IL-17 response for protection, such as fungal keratitis (40) and *Aspergillus-*triggered asthma (41). Further research is needed to determine the influence of Dcir in the IL-17 response of these models.

Nevertheless, Troegeler et al. demonstrated that *Clec4a2* ^—/—^ mice are prone to developing T_H_1 responses in a mycobacterial infection model (42), supporting a role for Dcir in shaping adaptive responses. Chronic fungal infections, such as paracoccidioidomycosis, which slowly evolve and are characterized by organ fibrosis due to the long-term accumulation of immune-driven tissue damage (43), could be affected by the potential of Dcir to shape this branch of the immune system.

Modulators of the immune response, such as Dcir, function through mechanisms maintain the host’s integrity during constant stimulation. Daily exposure to *A. fumigatus* spores can cause neutrophil overactivity, compromising lung homeostasis and increasing the risk of acute respiratory distress syndrome (44). Furthermore, Dcir deficiency predisposes individuals to non-infectious diseases, such as spontaneous autoimmune arthritis (13), experimental autoimmune encephalomyelitis/EAE (45), colorectal cancer (46), and atherosclerosis (47). Therefore, Dcir may participate in the regulation of immune system balance.

Consistent with a potential immunoregulatory function, the transient blockade of Dcir may serve as an adjunct therapy to improve antifungal defense in patients with aspergillosis. The use of soluble ligands, as the use of laminarin to block dectin-1 (48), is a possible strategy. However, the identity of Dcir ligand(s) remains poorly studied. While it has been proposed that this receptor recognizes conserved glycan motifs from host and exogenous sources (49), only host-derived molecules have been identified and structurally characterized [e.g., asialo-biantennary N-glycan in bone as reported by Kaifu et al. (10)]. Future research is necessary to clarify the clinical significance of these findings in humans and to identify those possible ligands in *A. fumigatus*, but our results suggest that the target molecules are readily accessible to the receptor and can be found in both conidia and hyphae.

This study has certain limitations. First, immunocompetent mice were used, whereas human patients are often immunosuppressed due to medication or the disease. Second, the inoculation doses do not reflect natural infections, though the model aligns with the standards established by other groups in the field (50–52). Translational research should consider these variables to further investigate the role of Dcir in the host response to *A. fumigatus.*

In conclusion, this study identified Dcir as a novel CLR family fungal sensor that regulates the antifungal response rather than promoting it. These findings broaden the understanding of CLR functions in host defense, highlighting additional complexity in host–fungi interactions.

## Data Availability

The original contributions presented in the study are included in the article/**Supplementary Material**. Further inquiries can be directed to the corresponding author/s.
