# Supplementary material for: The C-type lectin receptor Dcir (Clec4a2) restrains Aspergillus fumigatus elimination by limiting the degranulatory activity of neutrophils
Source: Front Immunol. 2025 Aug 4;16:1639400. doi: 10.3389/fimmu.2025.1639400 (PMC12358354; doi:10.3389/fimmu.2025.1639400)
Supplement: Supplementary file 1 [file DataSheet1.pdf]

SUPPLEMENTARY TEXT

Materials and Methods

Acute disseminated aspergillosis model

For the acute disseminated model, animals were inoculated by intravenous injection (lateral caudal vein) of 1 $x10^6$ conidia cells in 100 μL of PBS. On the designated days post-infection, mice were euthanized by cervical dislocation, and tissues were perfused with ice-cold PBS. Organs were harvested, weighed, and macerated in PBS. The suspensions were used for CFU and cytokine determinations.

SUPPLEMENTARY FIGURES

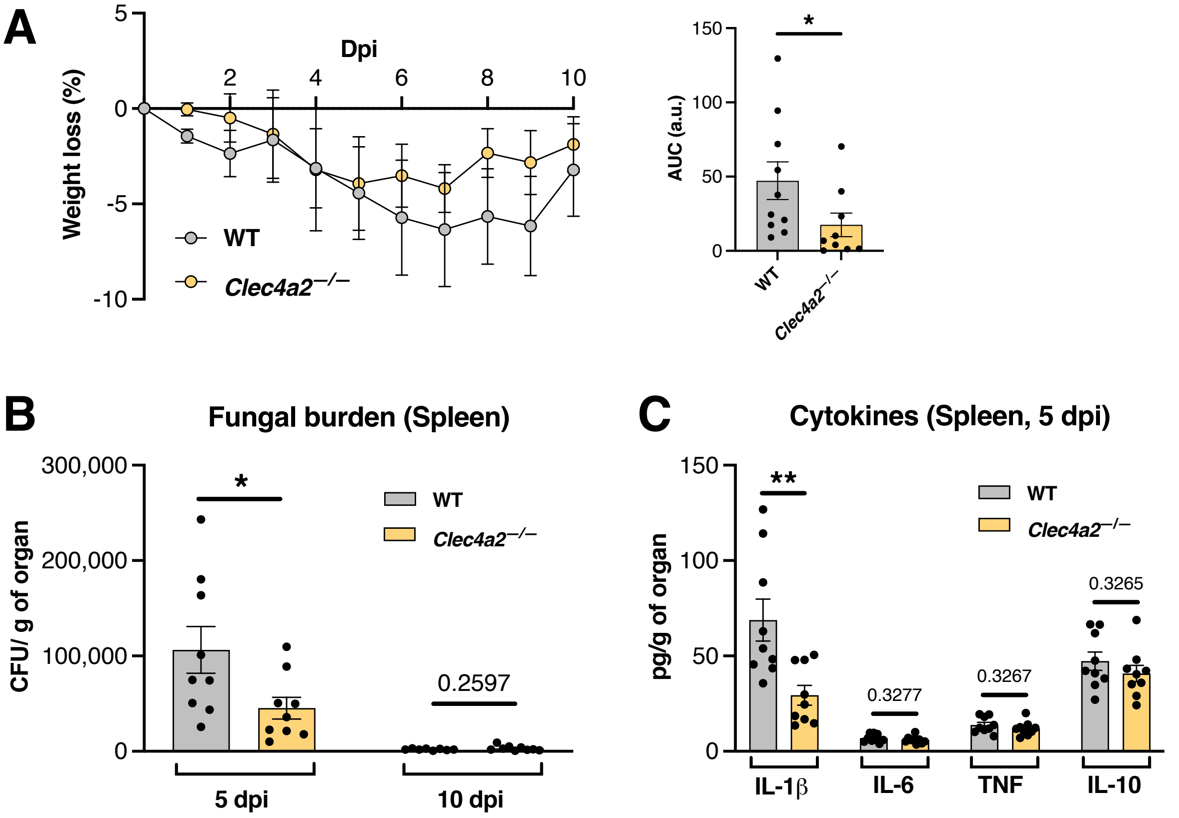

Supplementary Figure 1. Dcir-deficient mice show better resolution in the acute disseminated aspergillosis model. (A) WT and *Clec4a2*$^{-/-}$ mice were infected intravenously with 1 x10^6 *A. fumigatus* conidia and weight loss was monitored for 10 days post infection (dpi). (B) Fungal burden at 5 dpi and 10 dpi and (C) cytokine levels at 5 dpi in spleen macerates were determined. N = 9-10 mice per group, pooled from two independent experiments. Data are expressed as mean ± SEM (each dot represents one mouse). (A) and (C): Mann-Whitney test: * $p < 0.05$, ** $p < 0.01$. (B): Two-way ANOVA and Fisher's LSD test: ** $p < 0.01$.

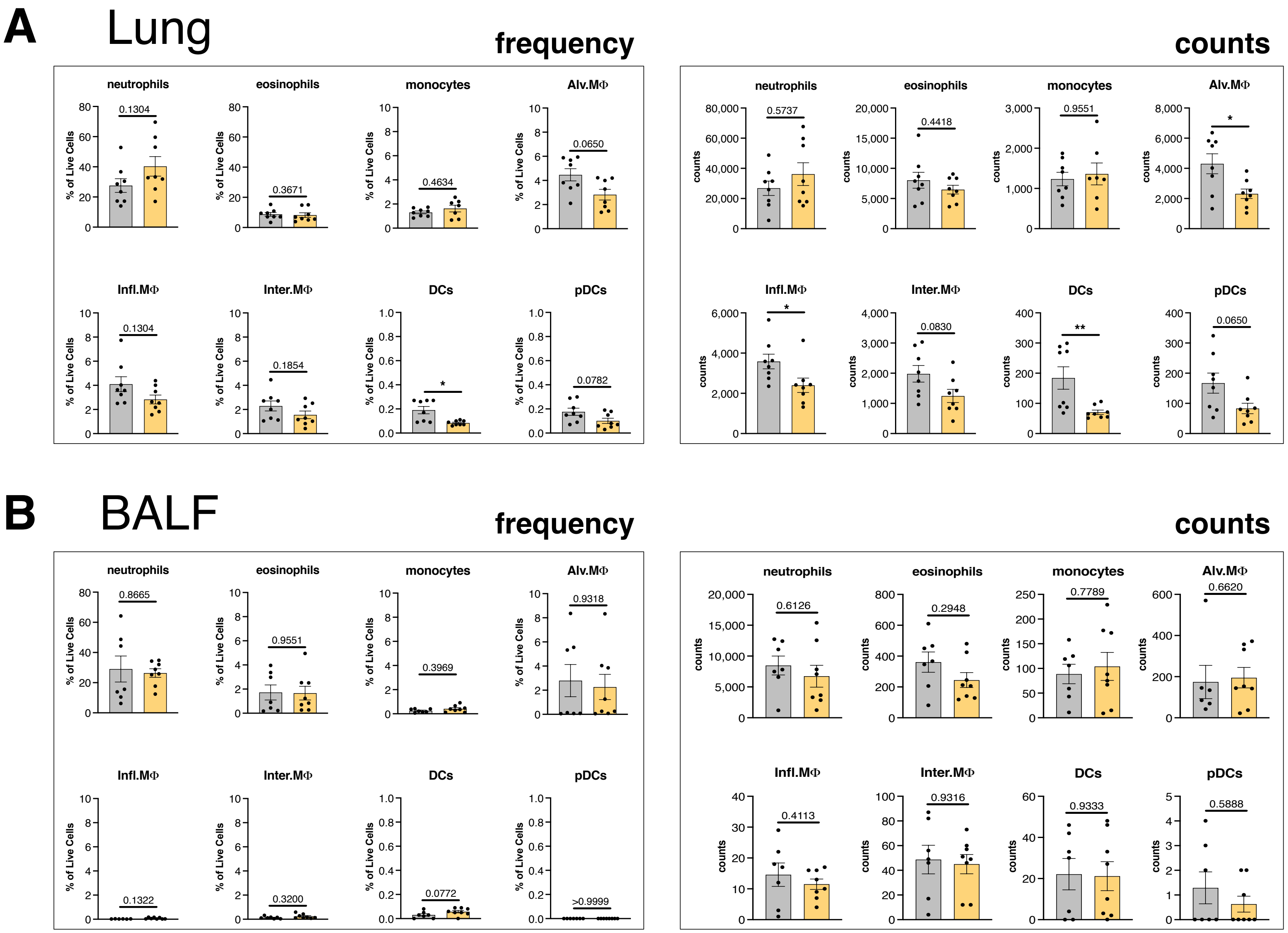
A
Lung
frequency
counts
neutrophils
eosinophils
monocytes
Alv.MΦ
Infl.MΦ
Inter.MΦ
DCs
pDCs
% of Live Cells
counts
0.1304
0.3671
0.4634
0.0650
0.1304
0.1854
*
0.0782
0.5737
0.4418
0.9551
*
*
0.0830
**
0.0650
B
BALF
frequency
counts
neutrophils
eosinophils
monocytes
Alv.MΦ
Infl.MΦ
Inter.MΦ
DCs
pDCs
% of Live Cells
counts
0.8665
0.9551
0.3969
0.9318
0.1322
0.3200
0.0772
>0.9999
0.6126
0.2948
0.7789
0.6620
0.4113
0.9316
0.9333
0.5888

Supplementary Figure 2. Profile of innate immune cells in the lungs and BALF of *A. fumigatus*-infected WT and *Clec4a2*$^{-/-}$ mice. WT and *Clec4a2*$^{-/-}$ mice were infected intratracheally with 1 x10^7 *A. fumigatus* conidia and samples were harvested at 5 dpi. Cell profile in (A) the lungs and (B) BALF was immunophenotyped by flow cytometry. *N* = 8 mice per group, pooled from two independent experiments. Data are expressed as mean ± SEM (each dot represents one mouse). Mann-Whitney test: * $p < 0.05$.

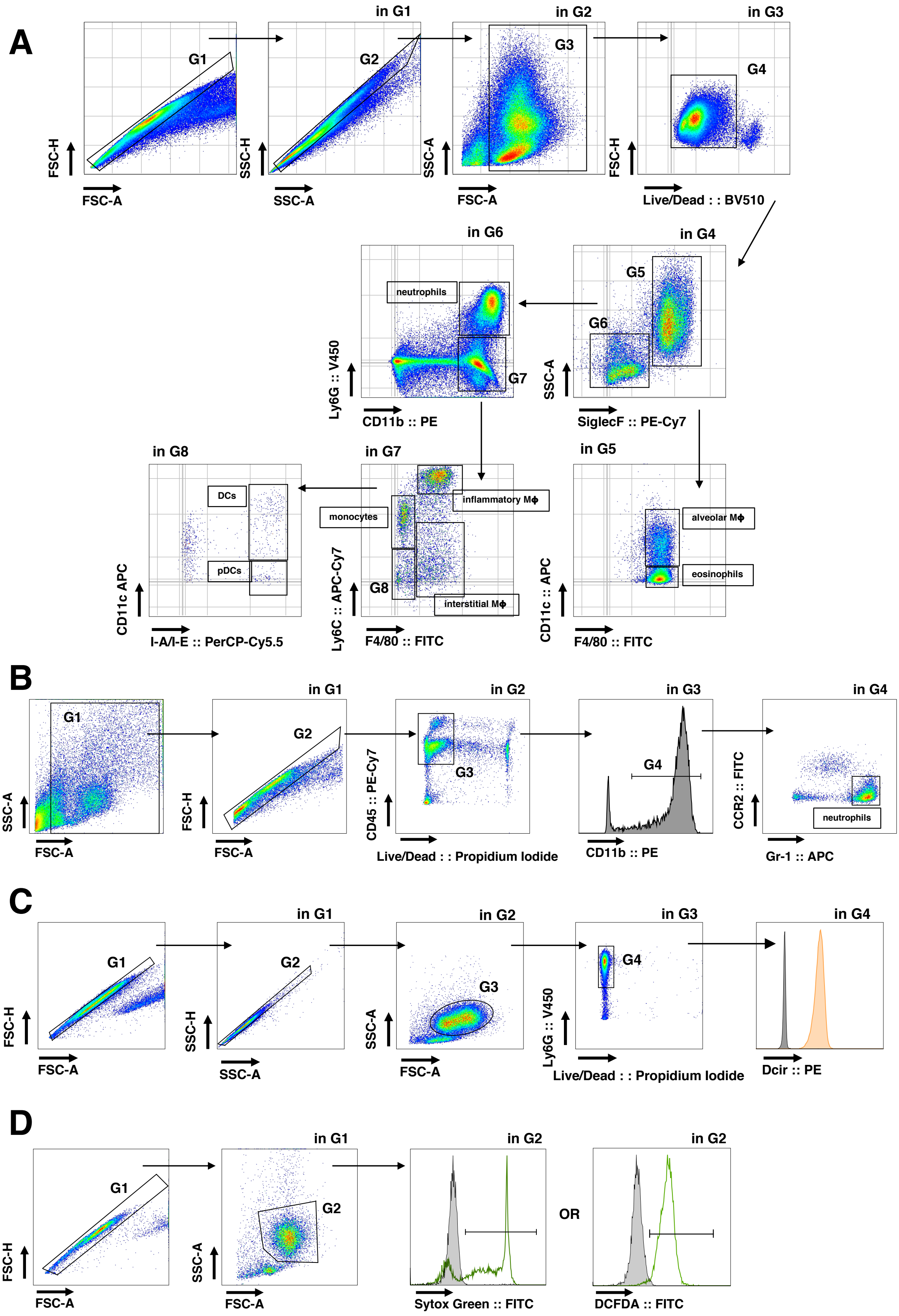
A
in G1
in G2
in G3
G1
G2
G3
G4
FSC-H
FSC-A
SSC-H
SSC-A
SSC-A
FSC-A
FSC-H
Live/Dead : : BV510
in G6
in G4
neutrophils
G5
G6
G7
Ly6G :: V450
CD11b :: PE
SSC-A
SiglecF :: PE-Cy7
in G8
in G7
in G5
DCs
pDCs
inflammatory Mϕ
monocytes
alveolar Mϕ
eosinophils
G8
interstitial Mϕ
CD11c APC
I-A/I-E :: PerCP-Cy5.5
Ly6C :: APC-Cy7
F4/80 :: FITC
CD11c :: APC
F4/80 :: FITC
B
in G1
in G2
in G3
in G4
G1
G2
G3
G4
SSC-A
FSC-A
FSC-H
FSC-A
CD45 :: PE-Cy7
Live/Dead : : Propidium Iodide
CD11b :: PE
CCR2 :: FITC
neutrophils
Gr-1 :: APC
C
in G1
in G2
in G3
in G4
G1
G2
G3
G4
FSC-H
FSC-A
SSC-H
SSC-A
SSC-A
FSC-A
Ly6G :: V450
Live/Dead : : Propidium Iodide
Dcir :: PE
D
in G1
in G2
in G2
G1
G2
OR
FSC-H
FSC-A
SSC-A
FSC-A
Sytox Green :: FITC
DCFDA :: FITC

Supplementary Figure 3. Representative gating strategies for the FCM analyses. (A) analysis of the cells in lung and BALF samples (related to Fig 2A and Fig S2). (B) analysis of the neutrophil influx in the BALF of anti-Ly6G antibody-depleted mice (related to Fig 2B). (C) analysis of the expression of Dcir in bone marrow-purified neutrophils (related to Fig 2C). (D) analysis of cell death or intracellular ROS in *in vitro* stimulated neutrophils (related to Fig 4B and Fig 4C).

SUPPLEMENTARY TABLE

Supplementary Table 1. List of reagents and antibodies for immunophenotyping. (in alphabetical order according to the target name)

**Reagent / Antibody**	**Clone**	**Manufacturer / Brand**
**CCR2** (CD192) FITC anti-mouse	SA203G11	Biolegend
**CD11b** PE anti-human/mouse	M1/70	Biolegend
**CD11c** APC anti-mouse	N418	Biolegend
**CD45** PE/Cy7 anti-mouse	30-F11	Biolegend
**DCIR1** (Clec4a2) PE anti-mouse	TKKT-1	BD Biosciences
**F4/80** FITC anti-mouse	BM8	Biolegend
Ly6G/Ly6C (**Gr-1**) APC anti-mouse	RB6-8C5	Biolegend
**I-A/I-E** PerCP/Cyanine 5.5 anti-mouse	M5/114.15.2	Biolegend
**Ly6C** APC/Cyanine7 anti-mouse	HK1.4	Biolegend
**Ly6G** violetFluor 450 anti-mouse	1A8	TONBObiosciences
**Propidium Iodide**	N/A	Sigma
PE Rat IgG2a, κ isotype Ctrl	RTK2758	Biolegend
**Siglec F** (CD170) PE/Cyanine7 anti-mouse	S17007L	Biolegend
Zombie Aqua™ Fixable Viability Kit	N/A	Biolegend
